# Reconocimiento de Emociones Básicas a Través de Expresiones Faciales en Niños con Trastorno por Déficit de Atención/Hiperactividad

**DOI:** 10.31083/RN37508

**Published:** 2025-06-25

**Authors:** Adriana Noelia Lewtak, Rocio González, Jesica Brenda Custodio, Amada del Rocio Mosquera Moncayo, Fernanda Rios Pistoia, Esteban Vaucheret Paz

**Affiliations:** ^1^Servicio de Neurología Infantil, Hospital Italiano de Buenos Aires, C1181ACH Ciudad de Buenos Aires, Argentina; ^2^Facultad de Psicología, Universidad de Buenos Aires, C1053 Ciudad de Buenos Aires, Argentina; ^3^Consejo Nacional de Investigaciones Científicas y Técnicas (CONICET), C1414 Ciudad de Buenos Aires, Argentina; ^4^Instituto de Ciencias para la Familia, Universidad Austral, C1010AAZZ Ciudad de Buenos Aires, Argentina; ^5^Idea21 de la Universidad Empresarial Siglo 21, C1055 Ciudad de Buenos Aires, Argentina; ^6^Laboratorio Moncayo, CP 091901 Ciudad Daule, Ecuador

**Keywords:** emociones básicas y complejas, expresiones faciales, niños, reconocimiento de emociones, TDAH, basic and complex emotions, facial expressions, children, emotion recognition, ADHD

## Abstract

**Introducción:**

El trastorno por déficit de atención/hiperactividad (TDAH) es un trastorno del neurodesarrollo que compromete funciones atencionales y ejecutivas, que pueden interferir en el reconocimiento de emociones faciales. En este estudio se exploró el reconocimiento de emociones básicas y complejas en sujetos pediátricos con TDAH.

**Método:**

Investigación de tipo prospectiva, transversal, controlada. Se incluyeron 60 participantes, divididos en 2 grupos: Grupo TDAH (n = 30) y el Grupo Control (n = 30) con desarrollo neurotípico. Se presentó a cada participante una serie de fotografías y videoclips de niños/as y adultos, debiendo identificar la emoción que se expresaba en el rostro.

**Resultados:**

No se hallaron diferencias significativas en el reconocimiento de emociones básicas entre el Grupo Control (M = 44,43; DS2,01), y el Grupo TDAH (M = 43,90; DS2,14; *t*(58) = –0,995; *p* = 0,324), ni en el reconocimiento de emociones complejas [*t*(58) = 0,514; *p* = 0,609]. No se encontraron diferencias por edad [*Z* = 463; *p* = 0,843] ni por sexo (*p* = 0,92). Observamos un rendimiento significativamente mejor con un elevado tamaño de efecto ante rostros infantiles (M = 29,56; IC 95% 28,98–30,14) en comparación con rostros adultos (M = 14,86; IC 95% 14,46–15,26; *p* < 0,001; d = 11,03), mejorando en estos últimos con edad (rho = 0,39; *p* = 0,03).

**Conclusión:**

El Grupo TDAH no muestra un desempeño diferencial respecto al Grupo neurotípico en el reconocimiento de emociones. El rendimiento mejora significativamente con rostros pediátricos por lo que deben evitarse rostros de adultos para evaluar a esta población. El reconocimiento en rostros adultos mejora con la edad.

## Abstract (en)

**Introduction:**

Attention-deficit/hyperactivity disorder (ADHD) is a neurodevelopmental disorder that affects attentional and executive functions, which may interfere with facial emotion recognition. This study explored the recognition of basic and complex emotions in pediatric subjects with ADHD.

**Method:**

This was a prospective, cross-sectional, controlled study. A total of 60 participants were included, divided into two groups: the ADHD Group (n = 30) and the Control Group (n = 30) with neurotypical development. Each participant was presented with a series of photographs and video clips of children and adults and was asked to identify the emotion expressed on the face.

**Results:**

No significant differences were found in the recognition of basic emotions between the Control Group (M = 44.43; SD = 2.01) and the ADHD Group (M = 43.90; SD = 2.14; t(58) = –0.995; *p* = 0.324), nor in the recognition of complex emotions [t(58) = 0.514; *p* = 0.609]. No differences were found by age [Z = 463; *p* = 0.843] or by sex (*p* = 0.92). We observed significantly better performance with a large effect size when recognizing child faces (M = 29.56; 95% CI 28.98–30.14) compared with adult faces (M = 14.86; 95% CI 14.46–15.26; *p* < 0.001; d = 11.03), with performance on adult faces improving with age (rho = 0.39; *p* = 0.03).

**Conclusion:**

The ADHD Group did not show differential performance compared with the neurotypical group in emotion recognition. Performance significantly improved for child faces, suggesting that adult faces should be avoided when assessing this population. Recognition of adult faces improved with age.

## 1. Introducción

El trastorno por déficit de atención/hiperactividad (TDAH) es uno de los trastornos del neurodesarrollo más frecuentes en la población infantil, con una variabilidad en el rango de prevalencia a nivel mundial que oscila entre el 2 y 12 % [1, 2, 3]. Se lo ha definido como un trastorno de inicio en la edad pediátrica antes de los 12 años, caracterizado por un patrón persistente de falta de atención e hiperactividad e impulsividad inapropiada para el grado de desarrollo del niño [4], que resulta en un impacto funcional en múltiples entornos y repercute en la calidad de vida tanto de quien lo padece como de su familia [5].

En el aspecto neuropsicológico, la evidencia científica muestra una considerable heterogeneidad en las alteraciones cognitivas presentes en el TDAH, entre ellos frecuentemente se asocia con dificultades en los diversos componentes de las funciones ejecutivas (FFEE), destacando el déficit en el control inhibitorio, la memoria de trabajo, planificación, así como un compromiso en funciones atencionales [6, 7].

El TDAH generalmente se asocia con problemas de conducta, dificultades en el vínculo con sus pares, problemas académicos y sociales [2], junto a una baja tolerancia a la frustración e irritabilidad [8], teniendo un impacto negativo en diversos aspectos de la vida diaria [1, 2, 8] y en el desarrollo del individuo [9]. Así mismo, en los niños y niñas que padecen este trastorno se ha observado deficiencias en la cognición social, siendo específicamente el reconocimiento de emociones en rostros el déficit más reportado [10, 11, 12], lo cual tienen un impacto en las interacciones sociales.

Las emociones son parte de la vida de todas las personas en todos los contextos en donde se desenvuelven. Dentro del terreno afectivo, nos referimos a una emoción como un estado efímero, de una duración que, en general, ronda sólo unos pocos segundos, y suele desencadenarse por una experiencia, desempeñando un papel esencial en la función adaptativa, puesto que preparan al organismo para que este reaccione cuando se presentan cambios en el medio, transmitiendo la información que se deriva de esto, rápidamente y de forma eficaz . De este modo, las emociones traducen lo que ocurre en el entorno externo y/o interno; es decir, la información es percibida y usada para actuar, resultando útil para la subsistencia [13, 14]. Dado que en toda interacción social humana suelen mezclarse emociones y cogniciones, es oportuno citar una función fundamental denominada cognición social, entendida como un concepto multidimensional que involucra un conjunto de procesos psicológicos, conscientes y no-conscientes, que subyacen a las interacciones interpersonales, incluyendo habilidades como el reconocimiento de las emociones de los demás, la teoría de la mente, el razonamiento moral, y la empatía [15, 16, 17]. De esta manera es un componente fundamental en la adaptación humana, permitiendo percibir, procesar, interpretar la información social y en consecuencia responder adecuadamente a cada situación [18].

El reconocimiento de emociones a través de la comunicación no verbal es uno de los procesos de cognición social más estudiados por ser una condición elemental para el éxito de la interacción social y la comunicación interpersonal [19, 20], ya que posibilita el poder reconocer, comprender y etiquetar verbalmente los estados emocionales a partir del procesamiento de señales emocionales que abarcan desde las expresiones faciales, el lenguaje corporal, hasta la información contextual [10, 21]. Específicamente el reconocimiento de emociones a través de las expresiones faciales, definida como la capacidad de percibir y analizar los rasgos faciales para la identificación del estado emocional que los mismos denotan, constituyendo un eslabón dentro de los componentes de la cognición social. En consonancia con esta línea teórica, Paul Ekman en la década del 70, realizó varias investigaciones sobre la conducta no verbal y el análisis de microexpresiones faciales, demostrando la universalidad de la expresión facial e identificó cierto tipo de expresiones faciales emocionales que denominó universales y básicas, por ser observables en diversas culturas [22, 23]. Estas emociones son seis: alegría, miedo, enojo, tristeza, sorpresa y asco, cada una de ellas acompañadas de una fisiología particular y de características faciales bien definidas, con valor adaptativo en la conducta social [24, 25, 26]. Por otro lado, hay otro componente que es aprendido, denominado “reglas de expresión” o de exposición incorporadas durante el proceso de socialización, que modulan ese mecanismo innato, por lo que no niega la existencia de otros movimientos corporales que pueden varían de una cultura a otra así como de ciertas diferencias en la forma de clasificar y expresar las emociones en las distintas culturas y entre sujetos pertenecientes a una misma cultura. Estas son reglas aprendidas culturalmente e internalizadas sobre qué emoción, durante cuánto tiempo, cuándo, cómo y con quién puede mostrarse [27]. Entonces, una expresión puede ser innata y universal y aun así mostrar una variabilidad individual y cultural referentes al significado semántico que se le atribuya.

Desde el punto de vista del comportamiento, las emociones humanas involucran múltiples componentes ya sean fisiológicos, perceptivos, expresivos, cognitivos y subjetivos, pudiendo ser expresadas mediante sonrisas, ceño fruncido y otro tipos de expresiones faciales. Por otra parte, algunos autores describen que dependiendo del significado que se les otorga en función del mayor o menor bienestar con el que se asocia, estas se las puede distinguir en emociones negativas, positivas y neutras. Las emociones negativas son aquellas que originan una sensación desagradable o aversiva sobre un evento como por ejemplo el miedo, tristeza, desagrado o enojo; las positivas, son generadoras de sensaciones agradables, como la alegría, y las neutras, son aquellas que no provocan interiormente reacciones ni agradables ni desagradables, como sucede con la sorpresa [14].

Aparte de las emociones básicas o primarias, se describen las “emociones secundarias o complejas”, que se trata de un amplio abanico de estados emocionales que nacen por combinaciones de las primarias, como por ejemplo, el resentimiento surge como parte de la combinación de tristeza y rabia o la decepción estaría compuesta por tristeza y sorpresa. Las emociones complejas tienen lugar a partir de la interacción social e implica atribuir un estado cognitivo y una emoción a otros, siendo más dependientes del contexto y la cultura, por lo tanto su reconocimiento requiere de una elaboración cognitiva del contexto social [27]. En ellas se suman, además, aspectos de otra naturaleza como las creencias, las intenciones o estados mentales que impactan de manera más amplia, por ello se considera que el reconocimiento de emociones secundarias se logra a través de la Teoría de la Mente. Las mismas se caracterizan por una variedad de respuestas, cambiando de una persona a otra, y no presentando un estilo de afrontamiento puntual y carecen de una forma de expresión facial específica. Estas emociones comienzan a identificarse a partir de los 7 años, señalándose como importantes para el funcionamiento social los siguientes: diversión, aburrimiento, decepción, vergüenza, celos, amabilidad, nerviosismo, indecisión y crueldad [28, 29].

En cuanto al desarrollo evolutivo del reconocimiento de emociones a través de expresiones faciales es considerado como un proceso complejo y progresivo que se desarrolla a lo largo de la vida desde sus inicios hasta la adultez, mejorando paulatinamente con la edad tanto el desempeño en velocidad y precisión como en la amplitud del vocabulario emocional, no obstante, el reconocimiento de emociones no es uniforme para todas las emociones, siendo la primera emoción en reconocerse la alegría, seguida de la tristeza, el enojo y el miedo [21, 28, 30].

Los niños con TDAH pueden presentar niveles de desarrollo diferente a nivel de la cognición social afectando tanto el comportamiento como las emociones. En esta línea, algunos hallazgos sugieren que independientemente de la forma de presentación del TDAH, estos muestran ser menos precisos para identificar emociones a través de la visualización de rostros sin contexto, requiriendo tiempos de reacción prolongados [31, 32], mientras que otros autores plantean que no presentan dificultades para procesar las señales afectivas y que las fallas en esta competencia social estarían afectadas por una falta de atención en lugar de una mala interpretación [33]. Otros estudios sostienen que las dificultades en el reconocimiento emocional no pueden explicarse por las disfunciones cognitivas características y propias del trastorno, sino a un fallo primario de la capacidad para codificar señales emocionales, con alteraciones específicas en los sistemas cerebrales que subyacen a las habilidades de procesamiento facial y que no hay asociación con el comportamiento impulsivo, la hiperactividad o coeficiente intelectual [12]. Finalmente desde otra postura en la literatura, mencionan que los niños con TDAH procesan adecuadamente las señales afectivas, no encontrando diferencias al compararlos con niños con desarrollo neurotípico, incluso con otras patologías tanto en el número de emociones correctamente identificadas como los tiempos de reacción, independientemente de que se encuentren bajo tratamiento farmacológico. Más aún, se reportó que las posibles causas de las deficiencias en el reconocimiento de emociones estarían relacionadas con la presencia de comorbilidades, como por ejemplo, el trastorno de conducta [12, 34].

De este modo, los estudios existentes exponen resultados diversos respecto al reconocimiento de emociones faciales en sujetos con TDAH. Por esta razón, en este estudio nos proponemos determinar eventuales diferencias en las respuestas entre emociones faciales básicas y complejas en individuos pediátricos con diagnóstico de TDAH. En función de la literatura, creemos que los resultados pueden aportar evidencias en relación a la comprensión del trastorno tendiente a su abordaje e intervención terapéutica más integral con el objetos de que puedan adquirir herramientas trascendentales para una mejor interpretación de la información social y en consecuencia responder con mayor eficacia a cada situación social.

## 2. Material y Metodos

### 2.1 Diseño

Se efectuó un estudio prospectivo de corte transversal con alcance analítico, de casos y controles.

### 2.2 Participantes

La muestra final estuvo conformado por 60 participantes, de sexo femenino y masculino. Se conformaron 2 grupos, el primero de ellos (Grupo TDAH) integrado por 30 participantes pediátricos con diagnóstico de TDAH en seguimiento por el Servicio de Neurología Infantil, que contaran con un diagnóstico realizado por un neurólogo especializado en trastornos del neurodesarrollo según los criterios del DSM-V (Diagnostic and Statistical Manual of Mental Disorders, Fifth Edition) y que contara con una evaluación neuropsicológica compatible con dicho diagnóstico. El segundo grupo (Grupo Control) estuvo compuesto por 30 participantes con un desarrollo neurotípico con un buen rendimiento académico, que asistían a instituciones educativas de la provincia de Buenos Aires.

Los criterios de inclusión fueron ser niños y niñas escolarizados, tener edades comprendidas entre 7 y 12 años, residir en Buenos Aires, presentar el consentimiento informado firmado por alguno de los padres y tener el asentimiento del participante.

En el grupo control se excluyeron a los sujetos que hubieran realizado alguna consulta con salud mental (psicología, psicopedagogía o psiquiatría) o neurología infantil en los últimos 5 años, además se excluyeron a quienes presentaron bajo rendimiento escolar, problemas de comprensión o comunicación, alguna discapacidad intelectual o algún otro trastorno informado por la Institución Educativa o la familia, así como a aquellos que no presentaron el consentimiento informado o asentimiento del niño, no completaron con ambas pruebas de reconocimiento de emociones y/o abandonaron la investigación. En el grupo TDAH se excluyeron a aquellos sujetos que presentaron alguna comorbilidad psiquiátrica (trastorno del estado de ánimo, trastorno de ansiedad, trastorno de conducta) o del aprendizaje (trastorno de la lectura, escritura o cálculo), así como quienes tuvieran alguna otra patología médica. Al momento de participar en este estudio los participantes no recibieron metilfenidato ni atomoxetina.

Los participantes fueron seleccionados por muestreo no probabilístico e intencional, y realizaron la tarea de forma individual y en modalidad online mediante plataforma ZOOM (https://www.zoom.com/, 6.4.6, Zoom Video Communications, Inc., San Jose, CA, USA), supervisados por alguno de los padres.

### 2.3 Instrumentos de Evaluación

#### 2.3.1 Test “The NIMH Child Emotional Faces Picture Set” (NIMH-ChEFS)

Es un conjunto de estímulos formados por imágenes digitales en color de alta resolución de caras emocionales de niños y niñas de 10 a 17 años de edad. Incluye 482 fotografías estáticas de rostros infantiles expresando las emociones de miedo, ira, alegría, tristeza y neutros con dos condiciones de mirada: directa y desviada [35].

#### 2.3.2 Test “NimStim Set of Facial Expressions” 

Es un conjunto disponible en color, con gran número de estímulos y de gran variedad de expresiones faciales, compuesto por 672 imágenes, con una amplia diversidad étnica al proporcionar fotografías estáticas de rostros adultos (mujeres y hombres) con características fisionómicas asiático-americanos, afroamericanos, europeo-americanos y latino-americanos. Cada uno de las identidades o modelos expresa 16 gestos faciales diferentes, incluyendo alegría, tristeza, asco, miedo, ira, sorpresa, neutralidad y calma, es decir, expresando una cara neutra con menos tensión muscular. Este set contiene versiones de boca abierta y cerrada de cada expresión [36].

#### 2.3.3 Test CAM-C en su Versión Argentina 

El test (Cambridge Mindreading Face - Voice Battery for Children) CAM-C en su versión original fue diseñado para medir el reconocimiento de emociones y estados mentales complejos (divertida/o, molesta/o, decepcionada/o, avergonzada/o, celosa/o, amorosa/o, nerviosa/o, indecisa/o, antipática/o) en sujetos pediátricos mayores de 8 años, a través de la expresión facial y vocal mediante dos tareas unimodales, una tarea de rostros y una tarea de voz. En su versión argentina solamente se utiliza una tarea de rostros, que comprende videoclips sin sonidos de actores pediátricos y adultos, representando emociones a través de la expresión facial, seleccionando 27 videoclips. Esta prueba evalúa 9 emociones complejas: mala humor, indeciso/a, celoso/a, decepcionado/a, amoroso/a, avergonzado/a, preocupado/a, nervioso/a, divertido/a [28, 37].

### 2.4 Procedimiento

Se seleccionaron los participantes pertenecientes al Grupo TDAH y Grupo Control, de acuerdo con los criterios de inclusión/exclusión. A partir de los datos obtenidos de la base de datos de sujetos con diagnóstico de TDAH en seguimiento por el Servicio de Neurología Infantil y que contaron con una evaluación neuropsicológica compatible con dicho diagnóstico y efectuada en la misma institución, se contactó a las familias participantes. Por otra parte, se contactaron a los niños y niñas del Grupo Control y sus respectivos padres por medio de las autoridades de las Instituciones Educativas.

Siguiendo los principios éticos vigentes, se les informó a los padres y sus hijos acerca de los objetivos de este estudio, la confidencialidad de los datos recolectados y se les aclaró que su participación era voluntaria, pudiendo abandonar la investigación en el momento que lo desearan. De esta manera, la participación de los niños fue voluntaria y sujeta al asentimiento de los mismos y al consentimiento informado de sus padres. Luego se procedió a la aplicación de los instrumentos de evaluación en forma individual, en modalidad online mediante plataforma ZOOM, supervisados por alguno de los padres. A fin de evitar la fatiga y/o agotamiento del participante, las pruebas, sin límite de tiempo predeterminado, se administraron en tres encuentros virtuales de extensión variable, pero que en la práctica tuvieron una duración media aproximada entre 20 y 30 minutos cada uno.

Para llevar a cabo la evaluación del reconocimiento de emociones básicas se seleccionaron en total 60 fotografías a color a partir de dos sets (uno con fotografías de niños y el otro de adultos): The NIMH Child Emotional Faces Picture Set (*NIMH-ChEFS*) y *NimStim* Set of Facial Expressions, incluyendo 37 fotografías de niños/as y 23 fotografías de adultos, con los cuales se evaluaron cuatro emociones básicas: ira, alegría, tristeza y miedo, según el modelo de Ekman. Del total de fotografías, se seleccionaron 4 fotografías para el ensayo de práctica: 1 hombre (alegría); 1 mujer (enojo); 1 niña (tristeza); 1 niño (miedo) y otras 56 fotografías para el desarrollo de la tarea. Para su administración, la tarea inicia con la presentación de las instrucciones y a continuación se realizaron cuatro ejercicios de práctica, una por cada emoción básica (enojo, alegría, miedo y tristeza). Las mismas fueron reproducidas en la pantalla de un ordenador, donde cada fotografía se acompañó de 4 opciones de respuestas (etiquetas emocionales) y el participante seleccionó aquella palabra que mejor describiera la emoción que expresaba el modelo en cada fotografía. Respecto a la puntuación se registró el número de aciertos obtenidos de emociones reconocidas correctamente, asignando un punto y cero puntos a cada error.

Por otra parte, para realizar la evaluación del reconocimiento de emociones complejas se aplicó el test CAM-C en su versión argentina y para su administración los videoclips fueron reproducidos en la pantalla de un ordenador. La tarea inicia con la presentación de las instrucciones y fueron seguidas por dos ejercicios de práctica, cada uno de los videoclip estuvo acompañada de 4 palabras (etiquetas emocionales) y el participante tuvo que elegir una opción como respuesta, que sea la que mejor describiera lo que siente la persona en cada videoclip. Para la puntuación se le asignó un punto a cada acierto y cero puntos a cada error.

Cabe destacar que en todas las tareas de reconocimiento de emociones no existió límite de tiempo para responder a cada uno de los ítems y la reproducción de las fotografías y los videoclips se realizaba una sola vez.

## 3. Resultados

Los 60 participantes se distribuyeron en 2 grupos. El Grupo TDAH con 30 participantes, en su mayoría masculinos (73,3%) con una edad media de 10,27 años (DE = 1,60). El Grupo Control con 30 participantes, en su mayoría femeninos (70%), presentaron una edad media de 10,37 años (DE = 1,52). Dado a que la edad es una característica fuertemente asociada con el reconocimiento de emociones, y debido a que la edad no mostró distribución normal, se realizó una prueba U de Mann-Whitney, no se encontrando diferencias estadísticamente significativas [*Z* = 463; *p* = 0,843].

El Grupo TDAH presentó una media de cociente intelectual total (CIT) de 89,58 (IC 95% 83,35–95,79). No encontramos correlación entre el CIT y el reconocimiento de emociones básicas (rho = 0.02; *p* = 0,93) ni entre el CIT y el reconocimiento de emociones complejas (rho = 0,24; *p* = 0,3). Así mismo, la edad de los participantes con TDAH no reveló una correlación con el reconocimiento de emociones complejas (rho = 0,23; *p* = 0,20), ni con el reconocimiento de emociones básicas (rho = 0.23; *p* = 0,21). Tampoco encontramos diferencias estadísticamente significativas en el reconocimiento de emociones complejas por sexo, obteniendo los varones una media de 16,77 (IC 95% 15,45–18,09) y las mujeres una media de 18.12 (IC 95% 14,53–21,7; *t*(28) = 0,97; *p* = 0,33). De igual manera, los varones con TDAH obtuvieron una media de reconocimiento de emociones básicas de 44,45 (IC 95% 43,48–45,41) y las mujeres una media de 44,37 (IC 95% 43,03–45,71; *t*(28) = 0,09; *p* = 0,92), no revelando diferencias estadísticas significativas.

Al analizar el tipo de estímulo, evidenciamos un mejor rendimiento en el Grupo TDAH, en forma estadísticamente significativa con un elevado tamaño del efecto, ante caras pediátricas con una media de 29,56 (IC 95% 28,98–30,14) en comparación con caras de adultos con una media de 14,86 (IC 95% 14,46–15,26; *t*(58) = 42,75; *p* < 0,001; d = 11,03). Así mismo no encontramos una correlación entre la edad y el rendimiento en el reconocimiento de emociones en caras pediátricas (rho = 0,09, *p* = 0,63), sin embargo el rendimiento tiene una correlación positiva con la edad cuando las caras son de modelos adultos (rho = 0,39; *p* = 0,03). Es decir, que los participantes mejoran su rendimiento en el reconocimiento de emociones básicas en rostros de adultos a medida que su edad es mayor.

Al analizar la cantidad de respuestas correctas por grupo en relación a el reconocimiento de emociones básicas, se observa que el Grupo Control obtuvo una media de 44,43 (DE 2,01), mientras que el Grupo TDAH presentó una media de 43,90 (DE 2,14; *t*(58) = –0,995; *p* = 0,324), que si bien es ligeramente menor, no revela una diferencia significativa estadísticamente.

En cuanto a las emociones complejas, estudiadas mediante la aplicación de la prueba psicométrica CAM-C en su versión argentina, pudimos observar nuevamente que el Grupo Control obtuvo una media de respuestas correctas de 16,70 (DE 3,18), ligeramente superior al promedio de respuestas correctos del Grupo TDAH 17,13 (DE 3,35; *t*(58) = –0,514; *p* = 0,609), lo cual si bien revela una tendencia, la misma no es estadísticamente significativa.

## 4. Discusión

Este estudio se propuso analizar el desempeño en el reconocimiento de emociones a través de las expresiones faciales en sujetos pediátricos con diagnóstico de TDAH, no encontrando diferencias en el reconocimiento de emociones básicas ni complejas entre este grupo de participantes y el grupo control compuesto por sujetos pediátricos con desarrollo neurotípico. No obstante, observamos que los participantes con TDAH mejoran su rendimiento cuando los rostros son de sujetos pediátricos, mejorando su desempeño con rostros adultos a medida que se acercan a la adolescencia y adultez.

Diversos estudios son consistentes con estos resultados. La investigación de Wells *et al*. (2019) [38] que incluyó a 64 niños, de una edad similar a la de nuestro estudio, de 8 a 13 años, divididos en dos grupos con y sin diagnóstico de TDAH, quienes se sometieron a pruebas de reconocimiento de emociones básicas mediante fotografías computarizadas de niños. Este estudio informó que los niños con TDAH requerían más tiempo para reconocer y responder a los estímulos, pero fueron igual de precisos que el grupo de desarrollo neurotípico, es decir, si bien los niños con diagnóstico de TDAH fueron más lentos en procesar la respuesta, no tuvieron mayores diferencias generales en la elección respecto de los controles [38, 39].

Por otro lado, otros trabajos sugieren un déficit significativo en la precisión del reconocimiento de emociones en sujetos pediátricos con TDAH. La investigación de Löytömäki *et al*. (2020) [40], realizó una comparación entre 50 niños, con una edad ligeramente diferente a la que empleamos en este trabajo, dado que presentaban entre 6 a 10 años con trastornos del neurodesarrollo con respecto a 106 niños del grupo control. A cada participante se le asignó tareas de reconocimiento de emociones mediante imágenes y videos, y una tarea en la que se tenían que emparejar las expresiones faciales y los tonos de voz, obteniendo como resultado que los niños con diagnósticos de TDAH, Trastorno del Espectro Autista (TEA) y Trastorno del desarrollo del Lenguaje (TDL) un rendimiento inferior al de los controles con desarrollo neurotípico en todas las tareas de reconocimiento de emociones básicas [40].

En otro trabajo previo publicado por nuestro grupo, en el cual se propuso identificar el perfil de reconocimiento de emociones en sujetos pediátricos con TDAH que incluyó participantes adolescentes hasta los 18 años, utilizando como estímulo fotos de niños y niñas. Se empleó una muestra de 56 participantes, divididos en dos grupos, 28 con TDAH y 26 en el grupo control con desarrollo neurotípico, a quienes se les aplicó el *Child Affective Facial Expression set* (CAFE). Se reportó que el grupo con TDAH presentó mayor dificultad en el reconocimiento de emociones en general y particularmente en las emociones de ira, sorpresa y expresión neutral [12]. A diferencia del estudio previo, en esta oportunidad, utilizamos otros estímulos faciales, incluyendo solamente 4 emociones (ira, miedo, alegría y tristeza), e incluso casi la mitad de ellos comprendieron caras de adultos; además de incluir el análisis de emociones complejas. Esta situación podría explicar las diferencias en los resultados obtenidos, dado que, los sujetos con TDAH presentaron un rendimiento significativamente mejor en el reconocimiento de emociones básicas de caras de modelos pediátricos. Así mismo, objetivamos que el rendimiento en el reconocimiento de emociones básicas en rostros de adultos mejoraba significativamente a medida de que los sujetos con TDAH eran más grandes. Esa situación concuerda con lo documentado en la literatura, dado que las personas encuentran mayor facilidad en el reconocimiento de caras y de emociones, en aquellos rostros que tiene familiaridad con aquellos a los que se exponen en su vida diaria. De esta manera es probable que a la población pediátrica le sea más fácil obtener mejores rendimientos en este tipo de tareas cuando los estímulos son del grupo etario al que pertenecen. Por otro lado, en el estudio actual, no tuvimos en cuenta las respuestas para cada emoción, lo que también puede explicar diferencias con el previo, más aún teniendo en cuenta que en este estudio se excluyeron emociones faciales de sorpresa que en el trabajo anterior revelaron diferencias entre los participantes con TDAH y los controles.

Si bien a la luz de los hallazgos de este estudio, los individuos con TDAH no presentan diferencias en el desempeño en el reconocimiento de emociones básicas y complejas a través de expresiones faciales, en comparación con los participantes neurotípicos, pudimos evidenciar que el desempeño fue significativamente mejor cuando los estímulos presentados corresponde a rostros pediátricos y que la capacidad de reconocer emociones básicas en rostros de adultos mejora con la edad, siendo mejor en sujetos mayores. En función de estos hallazgos se recomienda seguir investigando y profundizando al respecto, con muestras más amplias y diversas, incluyendo a las 6 emociones básicas faciales, seleccionando modelos pediátricos, así como con la incorporación de otras variables de estudio como comorbilidad con otro trastorno, el tiempo de respuesta.

Entre las limitaciones del estudio mencionamos el tamaño de la muestra, no considerar los tiempos o intervalos de respuesta, ni las respuestas correctas para cada emoción en particular. De ese modo, para futuras investigaciones se recomendaría incluir estas variables para profundizar en el estudio. Por último, para futuras líneas de investigación se sugiere incluir otras pruebas más ecológicas que incluyan otros componentes del lenguaje no verbal, como la prosodia y postura corporal en el reconocimiento de emociones.

Más allá de las limitaciones planteadas y teniendo en cuenta que en la mayoría de los estudios sobre la percepción y procesamiento de emociones en niños se ha basado en las respuestas de los niños a las caras de los adultos, la ventaja del uso de una prueba combinando dos set de fotografías para el reconocimiento de emociones básicas en esta investigación, reside en que es un conjunto grande de estímulos, de alta calidad, a color, digital y estandarizado de estímulos de emoción facial de los niños, favoreciendo así estas características fisonómicas el reconocimiento de las expresiones emocionales. Otro aporte importante fue haber aplicado una medida válida y confiable desarrollada localmente, el test CAM-C, en su versión argentina revisada, lo cual permitió evaluar el reconocimiento de estados afectivos complejos mediante estímulos faciales dinámicos que a su vez, se asemejan más a las demandas de la vida cotidiana.

Creemos que la profundización de estudios en esta línea deben efectuarse con rostros pediátricos y pueden aportar información trascendental que permita un mejor entendimiento sobre el reconocimiento de emociones en la evaluación de las personas con TDAH en la práctica clínica que permita establecer indicaciones terapéuticas más precisas.

## 5. Conclusiones

En nuestro estudio no encontramos diferencias en el reconocimiento de emociones faciales básicas ni complejas en sujetos pediátricos con TDAH. No obstante, observamos que los participantes con TDAH mejoraron su rendimiento cuando los rostros son de modelos pediátricos, por lo cual es aconsejable que en el futuro los estudios utilicen modelos de rostros pediátricos

## Data Availability

Los datos están disponibles a pedido.

## Contribuciones de los Autores

Investigación, curación de datos: AL, RG, EVP; Metodología, recursos, investigación, curación de datos: ARMM, RG, AL, FRP, EVP; Metodología, análisis formal: JC, AL, EVP; Metodología e investigación: AL, RG, EVP; visualización: JC, ARMM; RG; AL, FRP, EVP; Recursos y administración del proyecto: RG, AL, EVP. Todos los autores contribuyeron a los cambios editoriales del manuscrito. Mientras que la revisión crítica del contenido fue realizada por todos los autores, quienes aprueban la versión final del manuscrito y se hacen responsables de todos los aspectos del mismo, asegurando que las cuestiones relacionadas con la veracidad o integridad de todas las partes del manuscrito fueron adecuadamente investigadas y resueltas.

## Aprobación Ética y Consentimiento Informado

El estudio se realizó de acuerdo con las directrices de la Declaración de Helsinki y fue aprobado por el Comité de Ética del Hospital Italiano de Buenos Aires con el número 2894. Todos los sujetos o sus familias/tutores legales dieron su consentimiento informado para su inclusión antes de participar en el estudio.

## Agradecimientos

No aplicable.

## Financiación

Esta investigación no recibió financiación externa.
